# Research and application of bag filter system for railway ballast bed coal suction vehicles: An optimization and application study

**DOI:** 10.1371/journal.pone.0300192

**Published:** 2024-04-05

**Authors:** Ke Lu, Shengjun Guo, Zhongtai Zhao

**Affiliations:** 1 China Coal Technology and Engineering Group Chongqing Research Institute, Chongqing, China; 2 State Key Laboratory of Gas Disaster Detecting, Preventing and Emergency Controlling, Chongqing, China; NED University of Engineering and Technology, PAKISTAN

## Abstract

The current bag filter system used by railway ballast bed coal suction vehicles for cleaning coal dust from railway tunnels has low operational efficiency and generates significant volumes of dust. This paper describes a simulation test unit designed to enhance the dust removal performance in railway tunnels. The flow field inside the simulation test unit is investigated under different operating conditions through numerical simulations, and the variations in air volume and working resistance, total dust collection efficiency, and optimal operating parameters of a pulse cleaning system are identified through a series of experiments. The numerical results show that the pulse cleaning system does not significantly affect the uniformity of the flow field distribution at the bottom of the filter cartridge during the process of operation. The experimental research indicates that the simulation test unit satisfies the design requirements, achieving an average total dust removal efficiency of 99.93%. A field application shows that the total dust mass concentration at the operator position can be reduced from 335.8 mg∙m^−3^ to 4.2 mg∙m^−3^, effectively improving the operating environment within the tunnel.

## 1. Introduction

The main mode of coal transport in China is via railways [1]. The air disturbances generated as trains pass through tunnels cause large amounts of coal to spill out and accumulate on the railway line [2–5]. In some areas, the coal piles up flush with the railway track surface, which seriously endangers the safe operation of coal trains. With the increasing amount of coal transported by rail, such as on the Shenmu–Huanghuagang Railway, Baotou–Shenmu Railway, and Shuozhou–Huangshan Railway [6–8], this poses unacceptable risks to the safety of both trains and personnel.

For outbound transportation, railway coal line tunnels primarily rely on manual cleaning during window periods. The large amounts of coal piled up on both sides of the rails create uneven road surfaces in tunnels, making manual cleaning difficult and inefficient. Furthermore, manual cleaning leads to large volumes of coal dust particles floating within the tunnels, which pollute the operating environment; for example, the total mass concentration of coal dust in Shekoumao Tunnel was recorded at 335.8 mg∙m^−3^ during the cleaning process. Such high concentrations of coal dust seriously jeopardize the health of personnel and increase the risk of coal dust explosions [9]. Manual cleaning can instead be performed by railway ballast bed coal suction vehicles (CSVs). These vehicles offer improved cleaning efficiency, require fewer personnel, and ensure greater operational safety. The suction device and bag filter system are the two most important parts of a railway ballast bed CSV. The coal on the ballast in the tunnel is sucked in by the suction device and filtered by the bag filter system. However, the large amount of coal collected during railway tunnel cleaning frequently causes the filter element to become clogged, resulting in increased working resistance and reduced efficiency. Current designs for the bag filter system used on railway ballast bed CSVs lack theoretical guidance because there has been little research on the internal flow field distribution, dust removal efficiency, and pulse cleaning system. Thus, the existing bag filter system used by railway ballast bed CSVs falls short of the required effectiveness in railway tunnels.

Improving the coal suction efficiency of railway ballast bed suction vehicles has been widely studied. For example, Zhang [10] solved the problem of poor roadbed suction by increasing the speed of the blowing fan, optimizing the wind supply mode, and improving the structure of the blowing nozzle, effectively improving the suction performance. Zhan [11] analyzed the suction system of a miniature electric vacuum cleaner through computational fluid dynamics. The suction capacity was effectively improved by adopting the guiding and blowback method to maintain the fluid kinetic energy at the mouth of the nozzle. Hu et al. [12] designed a new dust suction device based on the particle initiation characteristics, and carried out numerical simulations of the flow field and experiments on the initiation mechanism of dust particles in different operating modes. Shi et al. [13] analyzed the dust suction mechanism of railway ballast bed CSVs and performed numerical simulations of the dust suction effect. Using computational fluid dynamics software, they simulated the internal flow field of a blowing suction device and developed improvements after analyzing the parameters affecting the dust suction efficiency. Although few nations have developed equipment especially for cleaning coal in tunnels, many countries have some technological expertise in the construction of equipment for vacuuming and cleaning trash along railway lines [14]. For instance, Switzerland has developed a dual-purpose tunnel dusting vehicle with an operating speed of 7 km/h. This vehicle achieves a good localized cleaning effect and is easy to use, but has a small storage capacity, which means it cannot be applied to long-distance tunnels with high levels of pollution. The SOCOFER and NEU companies in France and the Svolin company in Germany have also developed special vehicles for removing dust from tunnels. However, these vehicles typically have a small storage capacity, narrow cleaning range, and limited scope of application, and thus cannot achieve the high-efficiency coal suction capacity required for cleaning the railway tunnels along the coal transport lines of China [15, 16].

Existing research in China has mainly focused on improving the roadbed suction and dust removal system suction efficiency. However, research on the flow characteristics, dust collection efficiency, and pulse cleaning system of railway ballast bed CSVs is lacking. In this study, a simulation test unit was designed to enhance the main and side suction dust removal systems for railway ballast bed CSVs. The flow field inside the simulation test unit was investigated under different operating conditions through numerical simulations, while the variations in air volume and working resistance, total dust collection efficiency, and optimal operating parameters of the pulse cleaning system were examined experimentally. This paper describes the current problems and presents a method for improving the operational efficiency of the bag filter system used by railway ballast bed CSVs, thus providing theoretical guidance for the design of dust removal systems.

The rest of this paper is structured as follows: In Section 2, the railway ballast bed CSVs dust removal system was introduced, and the simulation test unit was designed. In Section 3, the numerical simulations research under different operation conditions is presented. In Section 4, the performance of the simulation test unit is evaluated by experimental research. In Section 5, the results of a field test conducted to verify the effect of the bag filter system for railway ballast bed CSVs are provided. Section 6 is the conclusion.

## 2. Railway ballast bed CSV dust removal system

**Fig 1:**
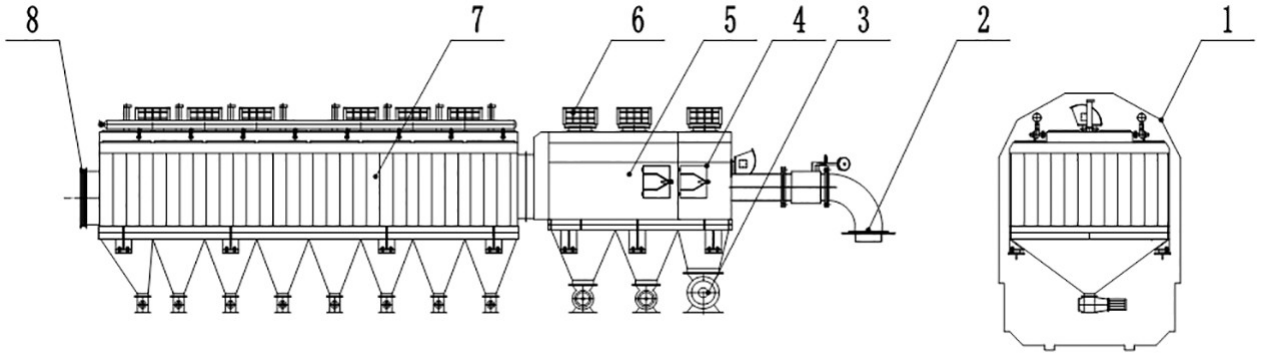
Schematic diagram of main suction dust removal system. 1- boundary of unit; 2- inlet of the airflow; 3- discharge valve; 4- gravity dust collector; 5- cyclone dust collector; 6- explosion venting device; 7- bag filter; 8- outlet of the airflow.

**Fig 2:**
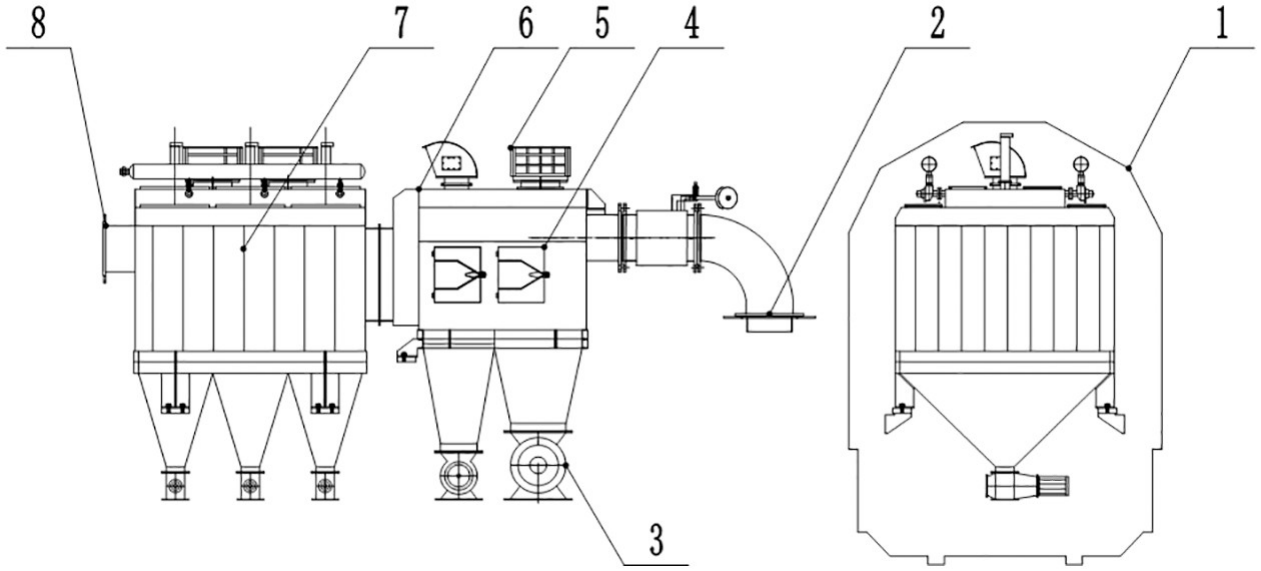
Schematic diagram of side suction dust removal system. 1- boundary of unit; 2- inlet of the airflow; 3- discharge valve; 4- gravity dust collector; 5- explosion venting device; 6- cyclone dust collector; 7- bag filter; 8- outlet of the airflow.

To collect coal particles from between and on either side of the tracks, a gravity dust collector, cyclone dust collector, and bag filter system are designed as one unit. The dust collection system is designed as a single-row arrangement to satisfy the size requirements of railway vehicles. One pulse cleaning system is designed to extend the service life of the bag filter system. The main and side suction dust removal systems are shown in Figs 1 and 2.

**Fig 3:**
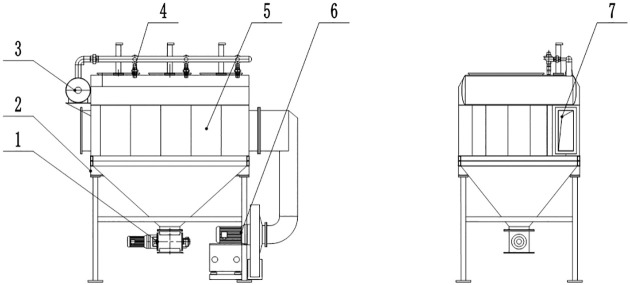
Schematic diagram of the simulation test unit. 1- discharge valve; 2- pedestal; 3- gas canister; 4- pulse valve; 5- main body of the test device; 6- exhaust fan; 7- inlet of the airflow.

The main and side suction dust removal systems of railway CSVs have the same dust removal principle, and the internal filter material and dust removal system are arranged in a circular way. Therefore, a simulation test unit was designed with reference to the main and suction dust removal systems. This unit was used to investigate the variations in air volume and working resistance, total dust collection efficiency, and optimal operating parameters of the pulse cleaning system. The simulation test unit is shown in Fig 3.

The simulation test unit has an air volume of 30 m^3^∙min^−1^, filter area of 11.6 m^2^, filter size of φ130 mm×600 mm, and fan total pressure of 3600 Pa. The pulse cleaning system is designed to prevent the filter cartridge from clogging, thus improving the efficiency of the bag filter system. The flow field space inside the simulation test unit is divided into different areas, and the valve switch of the corresponding area is controlled by an electric control system. When the different subchambers are closed, the corresponding valves are opened to release the compressed air, which blows on the filter cartridges of the different subchambers to clean the dust from their surface.

## 3. Numerical simulations

### 3.1. Determination of governing equations

In describing fluid flow, the law that should be followed first is the law of mass conservation. The law of mass conservation is the basic law that mass transfer should obey. In the process of fluid flow, no matter what flows from the fluid, the total mass of the flowing fluid should be constant. The simulated fluid was deemed to be of a steady flow and an incompressible fluid; thus, the density of the fluid was a constant [17]. Therefore, the law of conservation of mass was expressed as follows:

$$\frac{\partial \left(\rho \mu \right)}{\partial x}+\frac{\partial \left(\rho v\right)}{\partial y}+\frac{\partial \left(\rho w\right)}{\partial z}=0$$ (1)

where *ρ* is the fluid density; *t* is time; and *u*, *v*, and *w* are the projections of the velocity vector in *x*, *y*, and *z* directions, respectively.

The meaning of the law of conservation of momentum is that the action of the external force on the unit fluid micro-element will cause the fluid micro-element to generate momentum. The momentum of the fluid micro-element is equal to the rate of the change in the momentum of the fluid with time [18]. This explanation is often referred to as Newton’s second law. In the equations in which this is expressed, it is also often referred to as the N-S equation:

$$\left\{\begin{array}{l} \frac{\partial \left(\rho uu\right)}{\partial x}+\frac{\partial \left(\rho uv\right)}{\partial y}+\frac{\partial \left(\rho uw\right)}{\partial z}=\frac{\partial }{\partial x}\left(\mu \frac{\partial u}{\partial x}\right)+\frac{\partial }{\partial y}\left(\mu \frac{\partial u}{\partial y}\right)+\frac{\partial }{\partial z}\left(\mu \frac{\partial u}{\partial z}\right)-\frac{\partial p}{\partial x}+S_{u}\\ \frac{\partial \left(\rho vu\right)}{\partial x}+\frac{\partial \left(\rho vv\right)}{\partial y}+\frac{\partial \left(\rho vw\right)}{\partial z}=\frac{\partial }{\partial x}\left(\mu \frac{\partial v}{\partial x}\right)+\frac{\partial }{\partial y}\left(\mu \frac{\partial v}{\partial y}\right)+\frac{\partial }{\partial z}\left(\mu \frac{\partial v}{\partial z}\right)-\frac{\partial p}{\partial y}+S_{v}\\ \frac{\partial \left(\rho wu\right)}{\partial x}+\frac{\partial \left(\rho wv\right)}{\partial y}+\frac{\partial \left(\rho ww\right)}{\partial z}=\frac{\partial }{\partial x}\left(\mu \frac{\partial w}{\partial x}\right)+\frac{\partial }{\partial y}\left(\mu \frac{\partial w}{\partial y}\right)+\frac{\partial }{\partial z}\left(\mu \frac{\partial w}{\partial z}\right)-\frac{\partial p}{\partial z}+S_{w} \end{array}\right\}$$ (2)

where *ρ* is the density; *u*, *v*, and *w* are the projections of the velocity vector in *x*, *y*, and *z* directions, respectively; *μ* is the dynamic viscosity coefficient of the fluid; and p is the unit of the fluid micro-element. The pressure of the shared body and the meanings of *S*_*u*_, *S*_*v*_, and *S*_*w*_ are the generalized source terms of the momentum conservation equation.

The energy conservation equation [19] can be given as:

$$\frac{\partial \left(\rho T\right)}{\partial t}+div\left(\rho \mu T\right)=div\left(\frac{\text{k}\prime }{{\text{c}}_{\text{p}}}\text{gradT}\right)+S_{r}$$ (3)

where *T* is the ambient temperature, k′ is the heat transfer coefficient of the fluid, c_p_ is the specific heat capacity of the fluid, and *S*_*r*_ is the heat source in the fluid and the heat generated by the viscous action during the fluid flow.

The *k-ε* turbulence model is utilized to represent the wind flow’s trajectory because the wind flow flows erratically in the simulation test unit [20–25]. The equations for *k-ε* turbulence are:

$$\frac{\partial }{\partial \text{t}}\left(\rho k\right)+\frac{\partial }{\partial x_{i}}\left(\rho ku_{i}\right)=\frac{\partial }{\partial x_{j}}\left[\left(\mu +\frac{\mu_{\text{t}}}{\sigma_{k}}\right)\frac{\partial k}{\partial x_{j}}\right]+P_{k}-\rho \varepsilon$$ (4)

$$\frac{\partial }{\partial t}\left(\rho \varepsilon \right)+\frac{\partial }{\partial x_{i}}\left(\rho \varepsilon u_{i}\right)=\frac{\partial }{\partial x_{j}}\left[\left(\mu +\frac{\mu_{t}}{\sigma_{\varepsilon }}\right)\frac{\partial \varepsilon }{\partial x_{j}}\right]+C_{1\varepsilon }\frac{\varepsilon }{k}P_{k}-C_{2\varepsilon }\rho \frac{\varepsilon^{2}}{k}$$ (5)

$$\mu_{\text{t}}=\rho C_{\mu }\frac{k^{2}}{\varepsilon }$$ (6)

$$P_{k}=\mu_{\text{t}}S^{2}$$ (7)

$$S=\sqrt{2S_{ij}S_{ij}}$$ (8)

$$S_{ij}=\frac{1}{2}\left(\frac{\partial u_{i}}{\partial u_{j}}+\frac{\partial u_{j}}{\partial u_{i}}\right)$$ (9)

where *k* is the transport variable turbulent kinetic energy; *μ*_t_ is the viscous coefficient; *P*_*k*_ is the turbulent kinetic energy generated by the mean velocity gradient turbulent kinetic energy generated; *S* is the modulus of the mean strain rate tensor; *C*_*μ*_, *C*_1*ε*_, *C*_2*ε*_, *σ*_*k*_ and *σ*_*ε*_ is model constants, with default values of *C*_*μ*_ = 0.09, *C*_1*ε*_ = 1.44, *C*_2*ε*_ = 1.92, *σ*_*k*_ = 1, and *σ*_*ε*_ = 1.3.

The filter cartridge is porous medium, so the porous model is selected for simulation and set a boundary condition of porous medium, and the method of calculating permeability is in accordance with Darcy’s formula [26–28].

$$\Delta P=-\frac{\mu }{\alpha }v+{\text{C}}_{2}\frac{1}{2}\rho v^{2}\Delta m$$ (10)

where ΔP is the pressure loss, Pa; *μ* is the hydrodynamic viscous coefficient, Pa·s; α is the permeability, m^2^; *v* is the velocity component perpendicular to the surface of the filtration medium, m/s; C_2_ is the coefficient of pressure jump; Δm is the thickness of the filtration medium, m.

### 3.2. Geometry of modeling and grid generation

**Fig 4:**
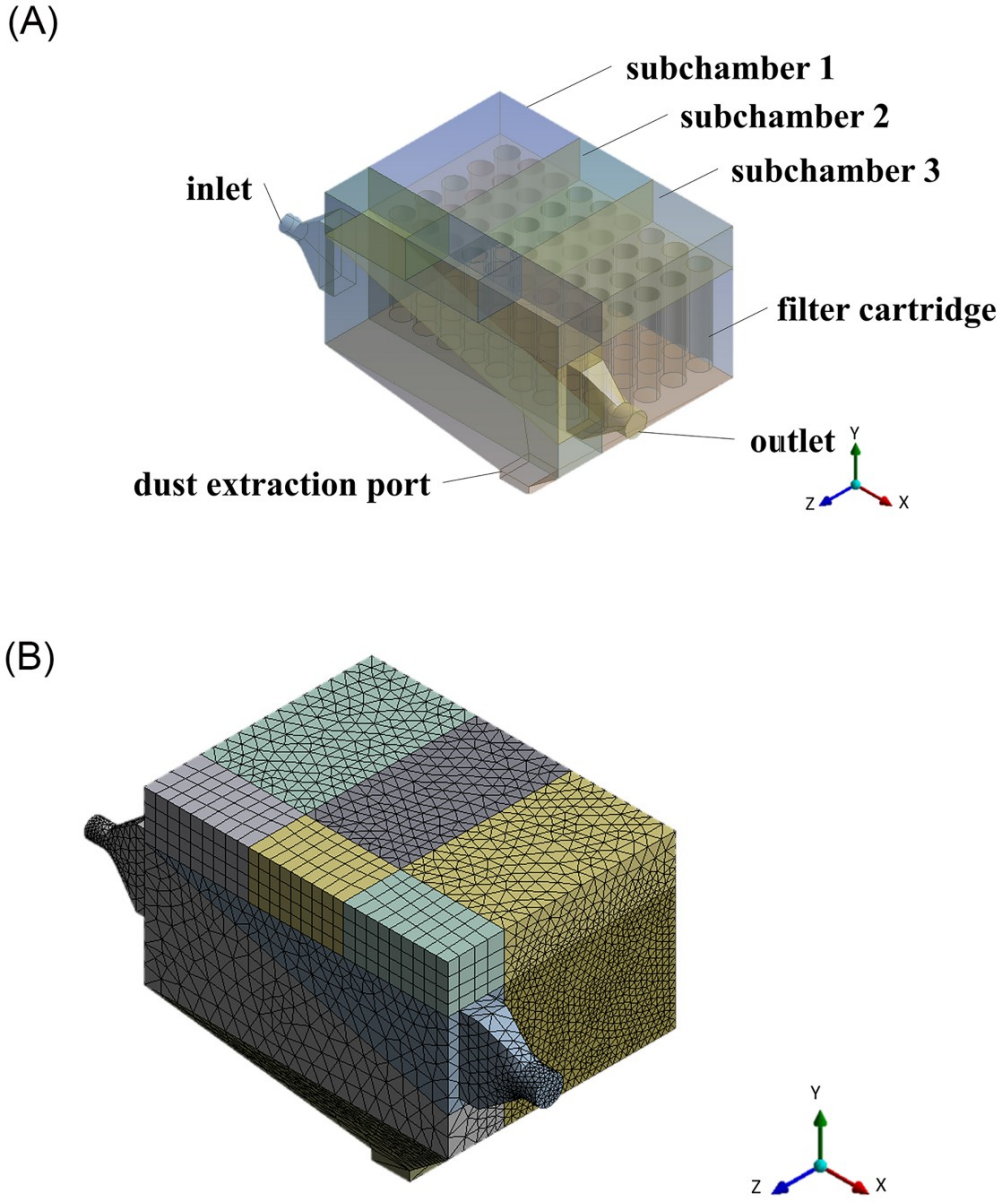
Schematic diagram of the numerical simulation model. (A) Schematic diagram of the model structure; (B) Schematic diagram of meshing.

The internal flow field of the simulation test unit was modeled using the Design Modeler software. The model was simplified by omitting structures that have a low impact on the flow field inside the simulation test unit from the modeling process. The grids for the model were generated using the Mesh software. The structure of the test unit and the mesh division are shown in Fig 4.

**Fig 5:**
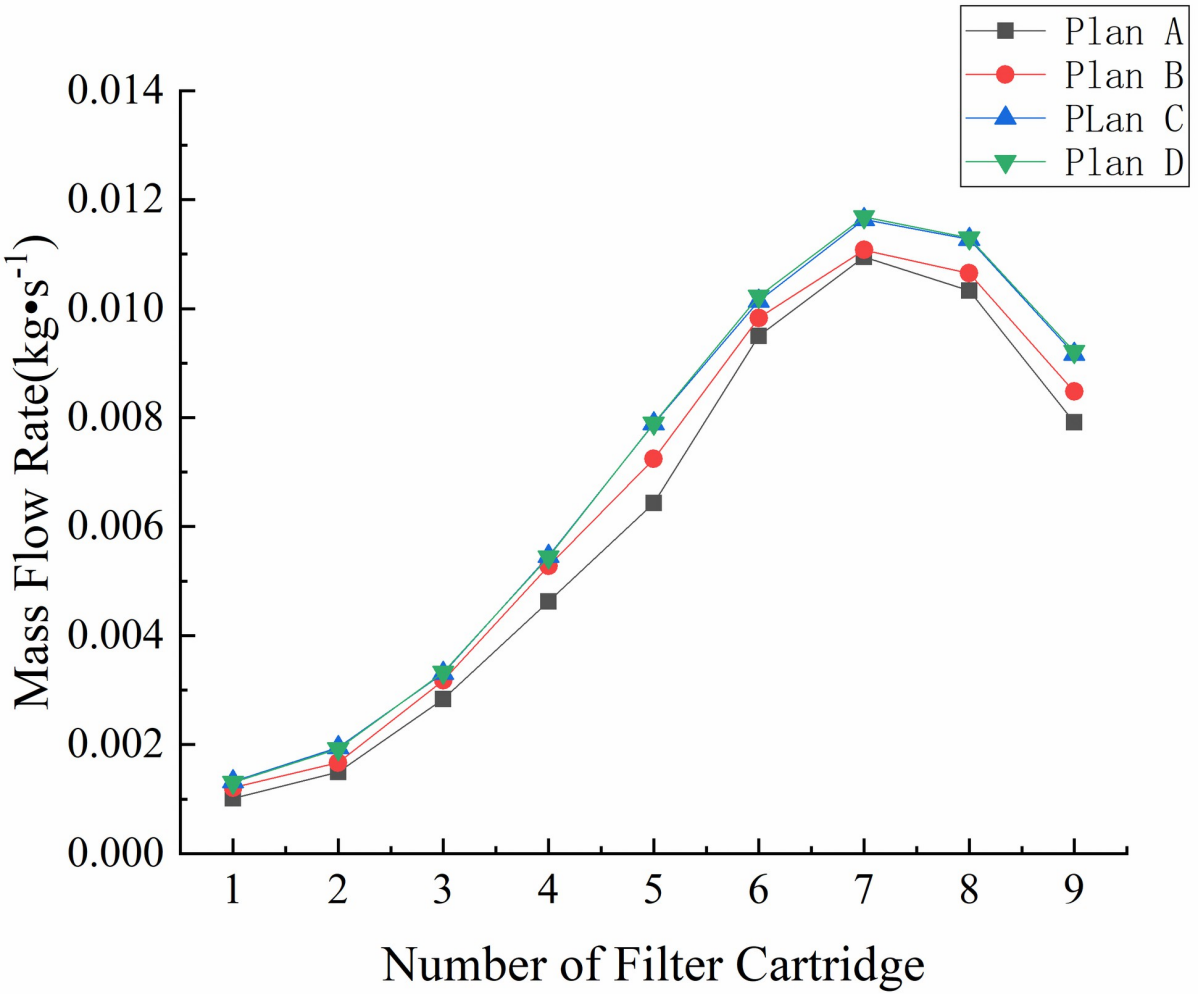
Variation in mass flow rate on the bottom surface.

The grid size has a significant influence on the accuracy and computational efficiency of a numerical model. Therefore, it is necessary to determine the appropriate mesh size for a specific problem. The model area was meshed using tetrahedral and hexahedral grid elements with four different resolutions. Mesh A had a total of 535,864 grid cells, mesh B had a total of 821,686 grid cells, mesh C had a total of 1,064,326 grid cells, and mesh D had a total of 1,328,210 grid cells. The flow fields of the grid models divided by the four mesh schemes were calculated under the same parameter settings. The variations in mass flow rate on the bottom surface of the nine selected cartridges are compared in Fig 5.

As can be seen from Fig 5, the mass flow rate at the bottom of the nine selected cartridges exhibits the same trend under all four mesh schemes. The difference in the mass flow rates given by meshes C and D is not more than 1.23%. Therefore, to ensure sufficient accuracy and computational efficiency, mesh C was selected for subsequent modeling.

### 3.3. Simulation parameters and boundary conditions

The model parameters and boundary condition settings are listed in Table 1.

**Table 1:** Parameter settings of numerical simulation model.

Name of Parameter	Value of Parameter
Inlet Type	PressureInlet
Inlet Pressure	0Pa
Wall Boundary Condition	No Slip
Outlet Type	MassFlowOutlet
Mass Flow	0.625 kg/s
Solution Method	The Pressure-Based Segregated Algorithm
Energy Equation	off
Porous Medium Model	on
Size of Filter Cartridge	φ130 mm×600 mm
Number of Filter Cartridge	45
Surface Permeability	9.08×e^−12^ m^2^
Thickness of Porous Medium	2 mm
Discretization Scheme	Second-order Upwind
Algorithm for Pressure-velocity	Coupled

### 3.4. Numerical simulation results

#### 3.4.1. Open subchambers

**Fig 6:**
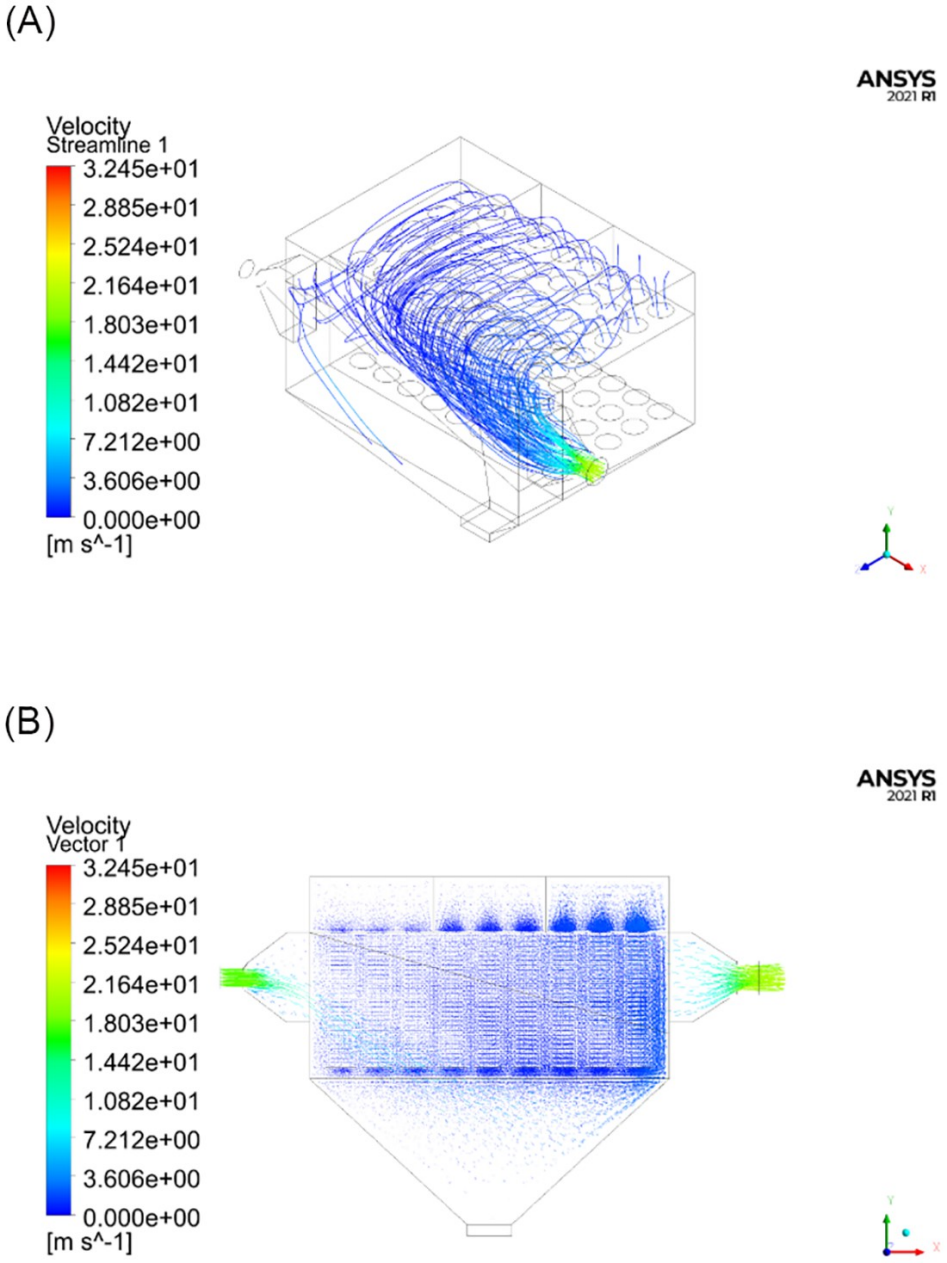
Numerical simulation results with open subchambers. (A) Schematic diagram of the streamline distribution; (B) Schematic diagram of velocity vectors.

Numerical simulations were conducted using the FLUENT software. The numerical calculations were performed on a computer with 32 GB memory and an i7-8700K 3.7 GHz CPU with 6 cores and 12 threads. The model parameters were set according to Table 1, and the numerical scheme terminated when the residual value was less than 1×10^−3^. A total of 873 iterations were required for the calculations to converge. The streamline distribution and velocity distribution of the flow field inside the test unit are shown in Fig 6.

#### 3.4.2. Closed subchambers

**Fig 7:**
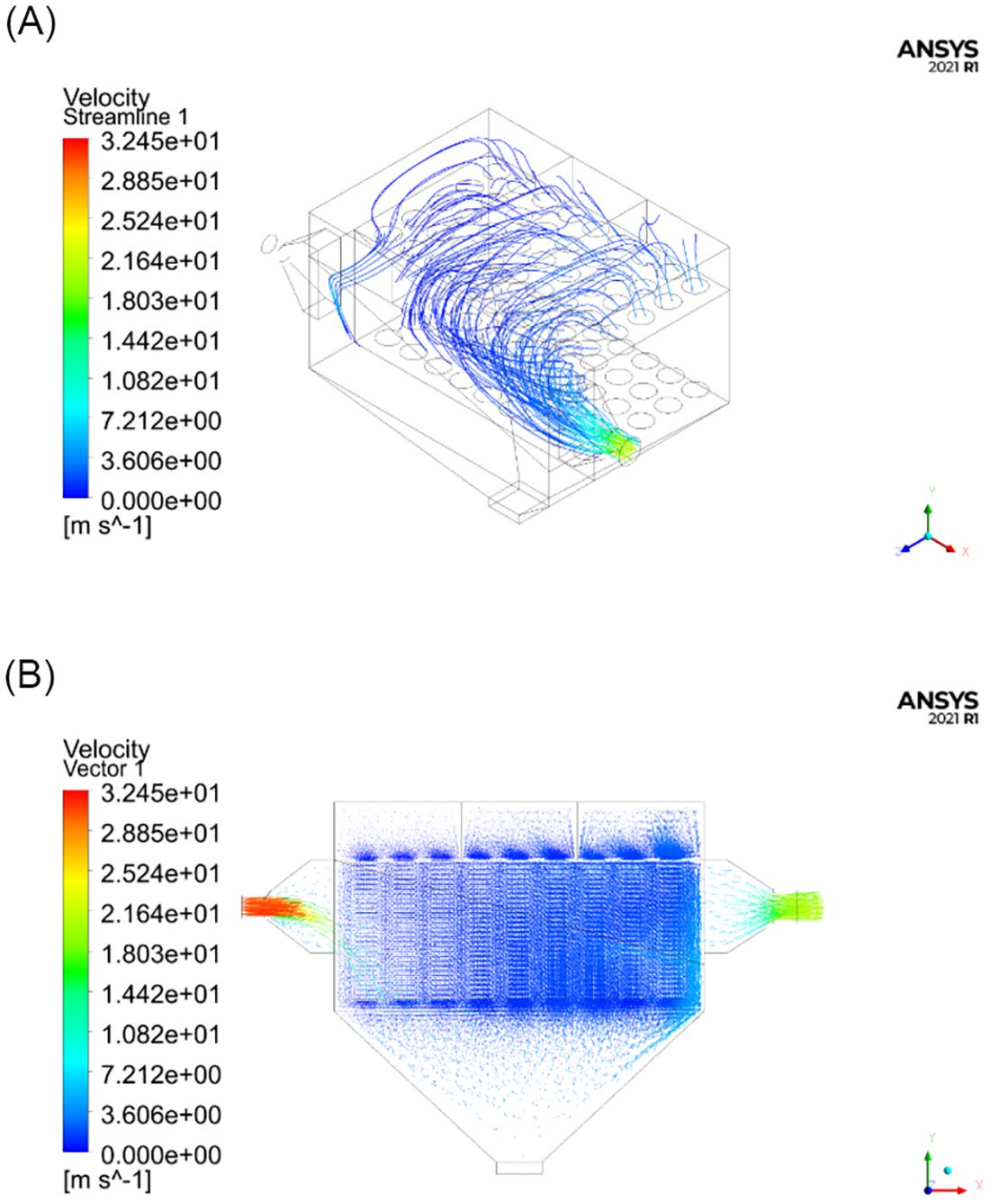
Numerical simulation results with subchamber 1 closed. (A) Schematic diagram of the streamline distribution; (B) Schematic diagram of velocity vectors.

**Fig 8:**
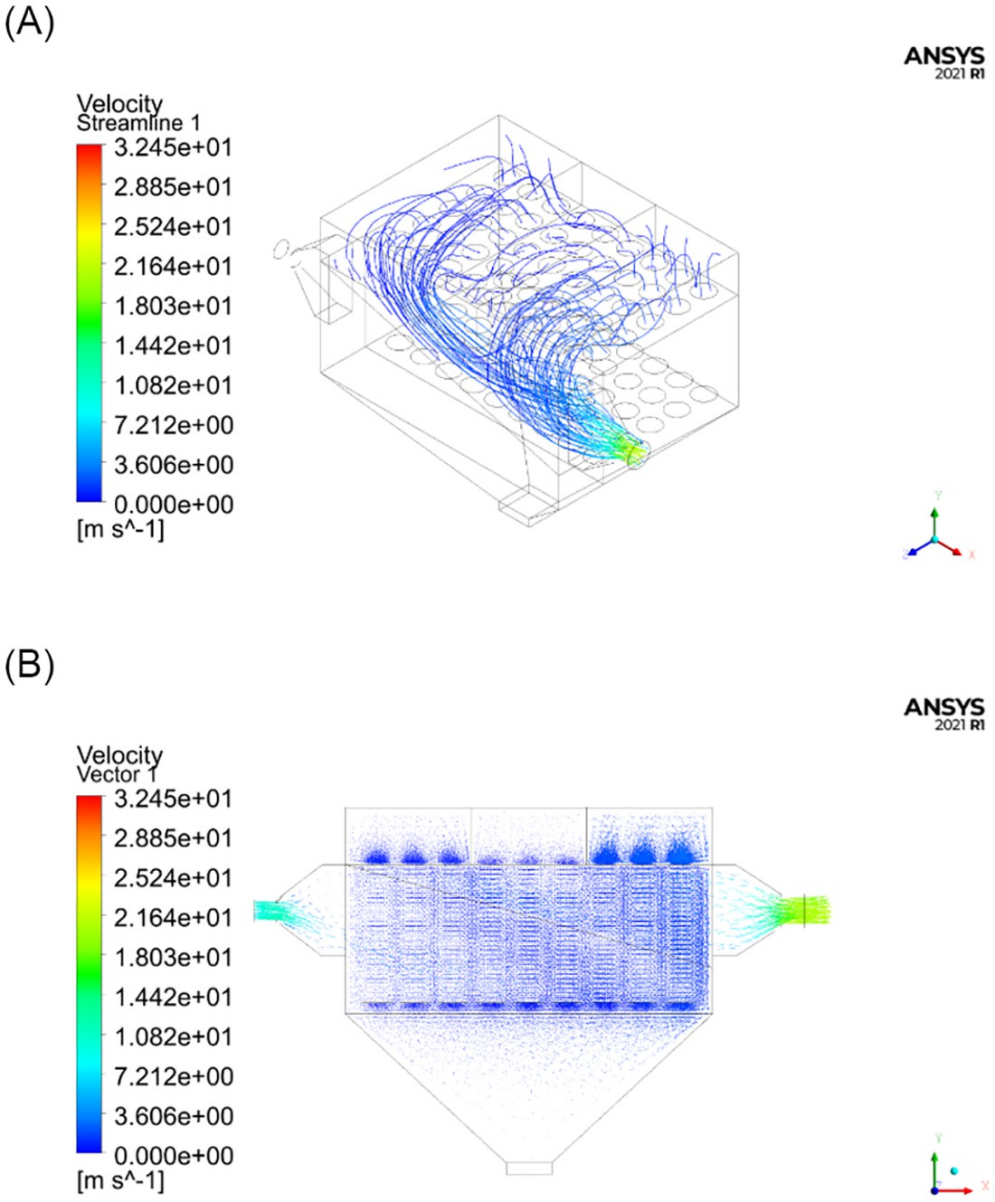
Numerical simulation results with subchamber 2 closed. (a) Schematic diagram of the streamline distribution; (B) Schematic diagram of velocity vectors.

**Fig 9:**
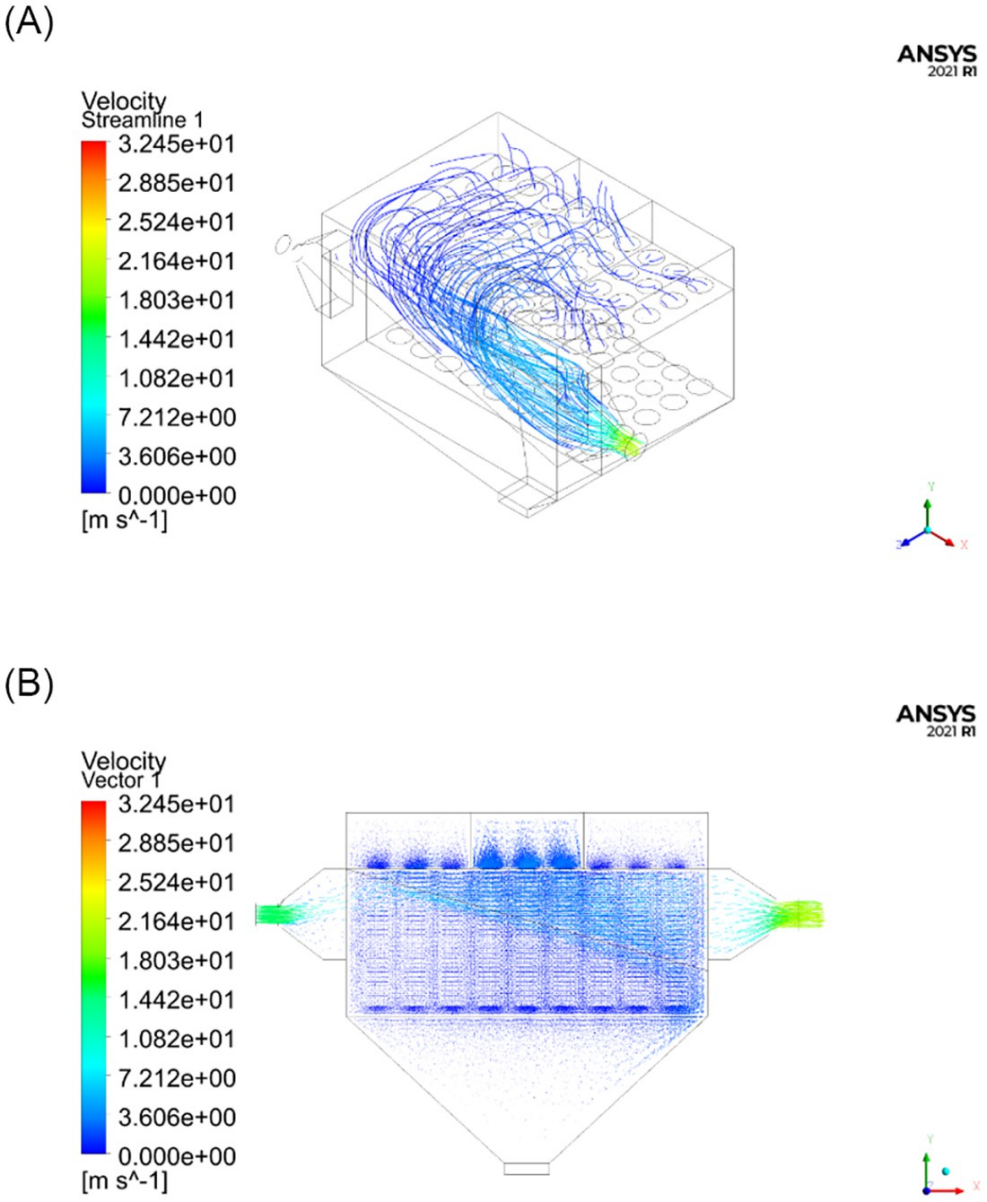
Numerical simulation results with subchamber 3 closed. (A) Schematic diagram of the streamline distribution; (B) Schematic diagram of velocity vectors.

The goal of this experiment was to simulate the changes in the flow field inside the test unit under three different operating conditions, namely with subchambers 1, 2, or 3 closed. The parameters of the model were set according to Table 1. The numbers of iterations required for the three different operating conditions were 836, 885, and 891, respectively. The numerical results are shown in Figs 7–9.

As can be seen from the simulation results in Figs 6–9, the distribution of the flow field inside the simulation test unit is affected by whether the subchamber valves are open or closed during the cleaning process. Under different operating conditions, the performance of the dust removal system varies. Consequently, the flow uniformity index was used to evaluate the uniformity of the internal flow field inside the simulation test unit.

### 3.5. Analysis of flow field uniformity inside test unit

The uniformity index is an evaluation standard for flow field velocity uniformity [29–34]. The calculation formula is:

$$r=1-\frac{1}{2n}\sum_{i=1}^{n}\frac{\sqrt{{\left(v_{i}-v\right)}^{2}}}{v}$$ (11)

where *r* is the uniformity index; *v*_*i*_ is the velocity at the observation point [m/s]; *v* is the average velocity across all observation points [m/s]; and *n* is the number of observation points. The uniformity index takes a value between 0 and 1, with higher values indicating a more uniform flow.

**Fig 10:**
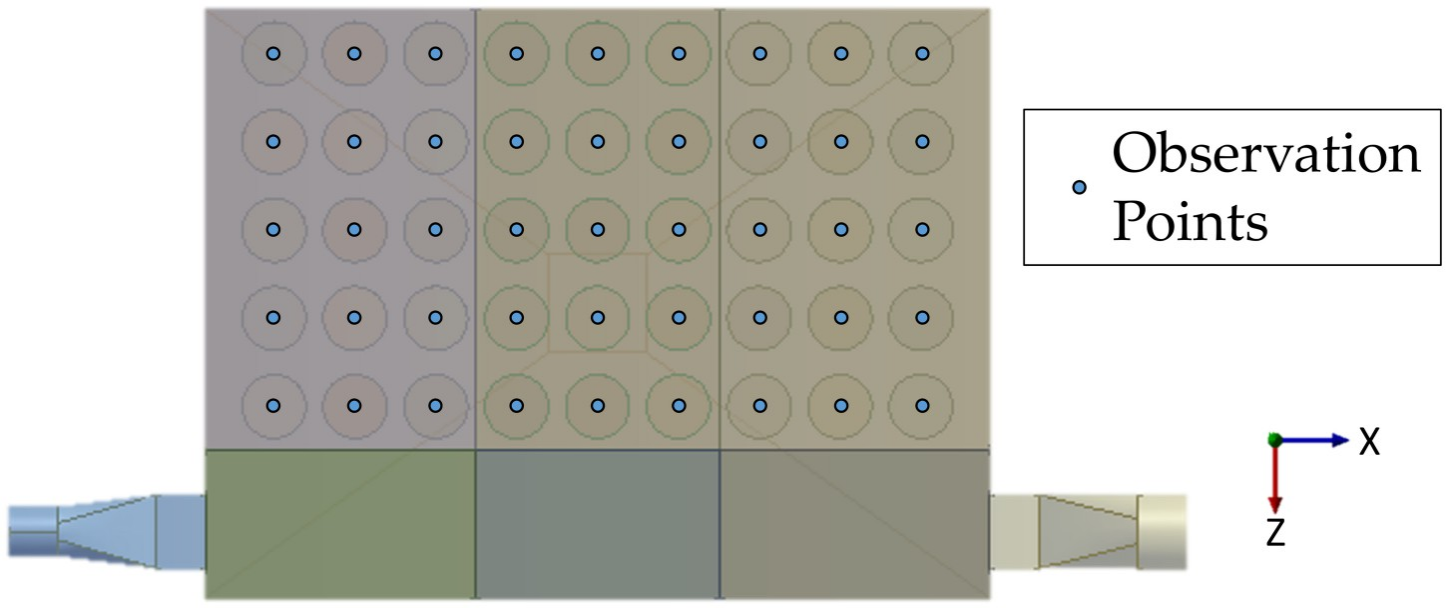
Layout of observation points.

The observation points were positioned 50 mm from the bottom center of the cartridge in the negative direction of the Y-axis. There were a total of 45 observation points; the arrangement of the observation points is shown in Fig 10.

**Fig 11:**
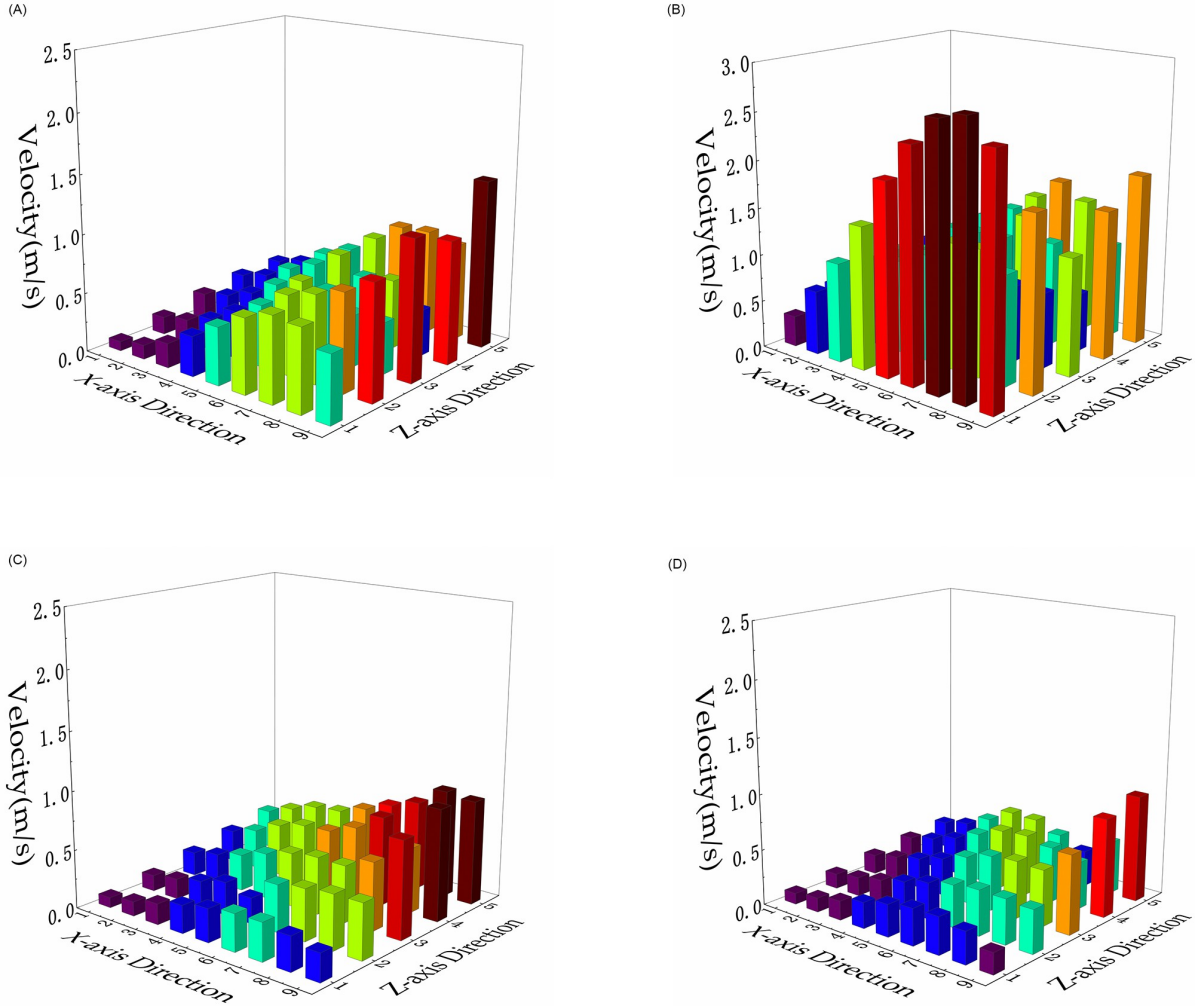
Histogram of speed values at measuring points under different conditions. (A) All subchambers open; (B) subchamber 1 closed; (C) subchamber 2 closed; (D) subchamber 3 closed.

The velocities at the observation points under the different operating conditions were exported using the CFD-Post module. Histograms of the results are shown in Fig 11.

The uniformity index was calculated using Eq (11). The results are presented in Table 2.

**Table 2:** Calculation results of uniformity index.

Operating Condition	Uniformity Index
Subchamber unclosed	0.795
Subchamber 1 closed	0.812
Subchamber 2 closed	0.781
Subchamber 3 closed	0.814

The calculation results show that the uniformity index is 0.795 when all subchambers are open. The uniformity index falls slightly to 0.781 when subchamber 2 is closed. The uniformity index takes values of 0.812 and 0.814 when subchamber 1 is closed and when subchamber 3 closed, respectively. Thus, the pulse cleaning system does not significantly affect the uniformity of the flow field distribution at the bottom of the filter cartridge in the simulation test unit.

## 4. Experimental research

### 4.1. Testing system

**Fig 12:**
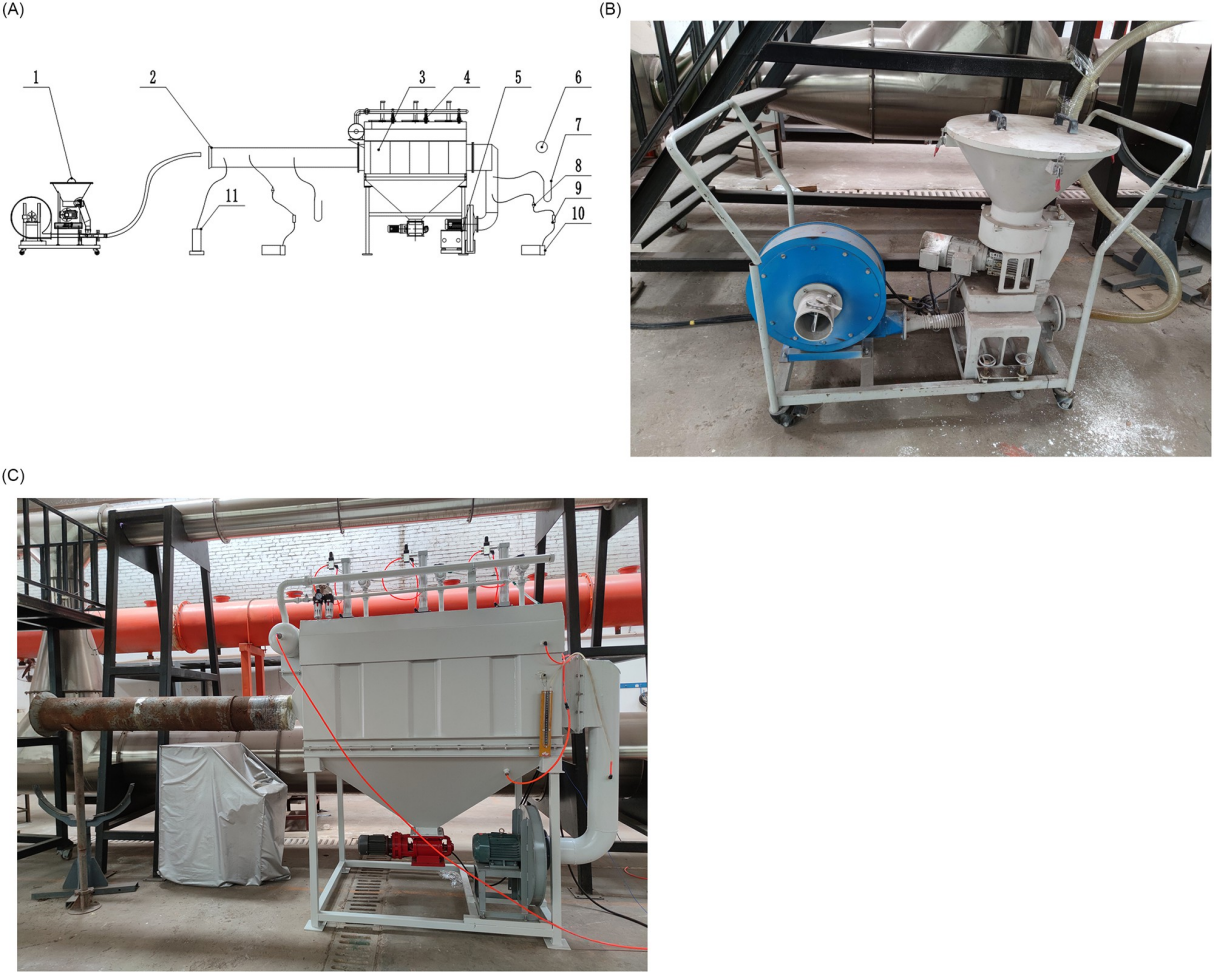
Diagram of test system and test devices. (A) Schematic diagram of test system: 1- quantitative dust generator; 2- air duct; 3- simulation test unit; 4- solenoid valve; 5- exhaust fan; 6- aneroid barometer; 7- U-type manometer; 8- sampling box; 9- rotameter; 10- vacuum pump; 11- compensated micro pressure gauge; (B) quantitative dust generator; (C) simulation test unit.

A test platform (Fig 12) was designed to evaluate the performance of the simulation test unit. Tests were conducted to determine the air volume, working resistance, total dust collection efficiency, and optimum parameters of the pulse cleaning system.

### 4.2. Air volume and working resistance

**Fig 13:**
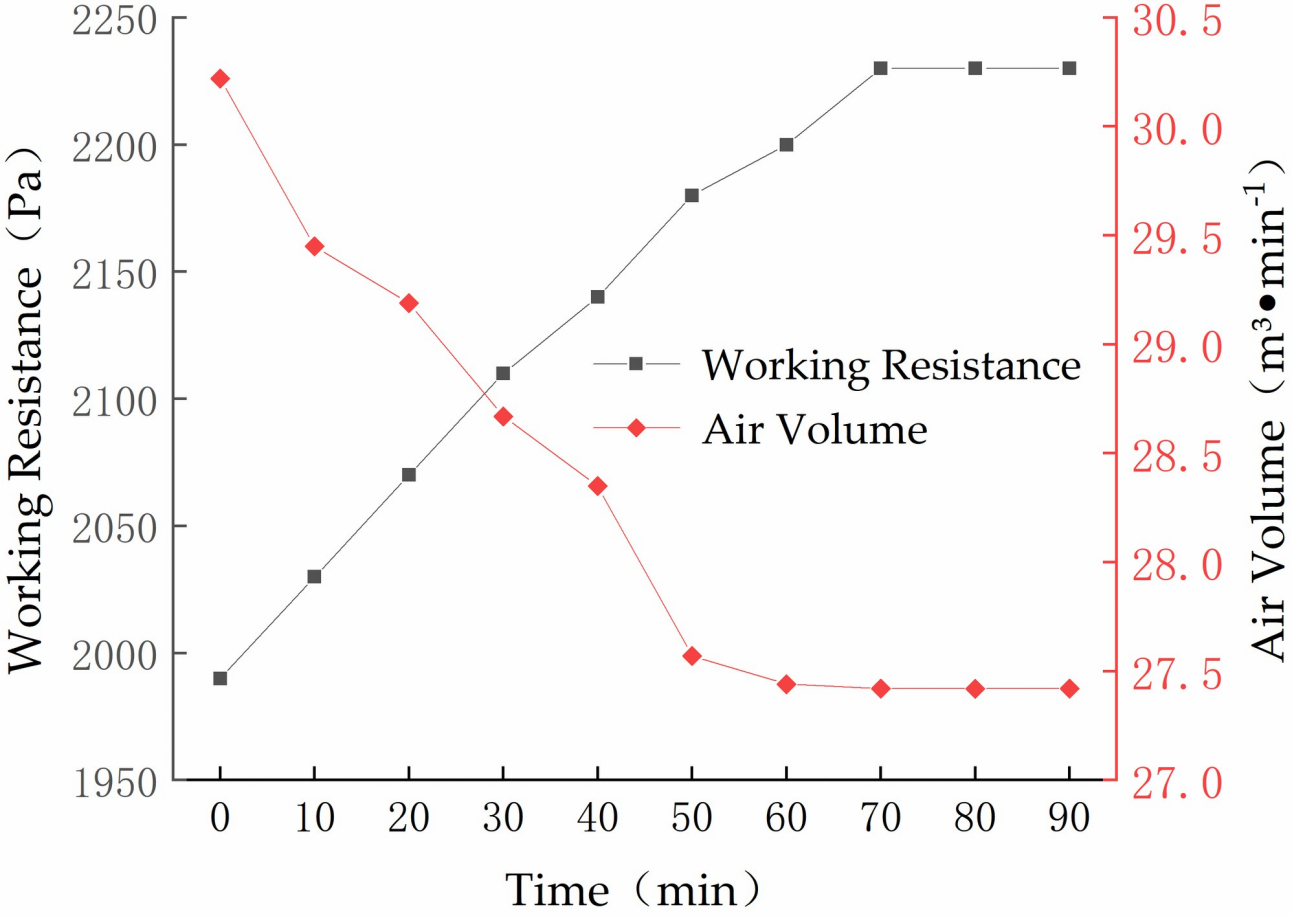
Variations in air volume and working resistance of the simulation test unit.

The working resistance of a dust collection is the pressure loss that occurs when air passes through the dust collection, which could affects the air volume of the dust collection. The working resistance of a bag filter is mainly influenced by its size, internal structure, number of filter elements, total filter area and other factors. Therefore, when designing a bag filter, it is necessary to study the variations in air volume and working resistance of the bag filter. The mass concentration of the quantitative dust generator was fixed to 1000 mg∙m^−3^. The variations in the air volume and working resistance of the simulation test unit are shown in Fig 13.

Fig 13 shows that the original working resistance and air volume of the simulation test unit were 1990 Pa and 30.22 m^3^·min^−1^. After the dust generator had been operating for 70 min, the working resistance had increased to 2230 Pa and the air volume had been reduced to 27.42 m^3^·min^−1^. The working resistance and the air volume remined stable thereafter.

### 4.3. Total dust collection efficiency

According to the test method for the total dust mass concentration (as detailed in China’s Universal Technical Specification for Dust Collection Enginery for Mine), the total dust removal efficiency was measured over a sampling time of 1 min at a sampling flow rate of 30 L/min. Dust removal efficiency can be calculated according to the formula (12) and (13):

Firstly, the dust mass concentration in the pre-treatment air and treated air is calculated by following formula:

$$c=\frac{m_{2}-m_{1}}{qt}\times 1000$$ (12)

where *c* is total mass connection, mg∙m^-3^; *m*_2_ is mass of the membrane after dust collection, mg; *m*_1_ is mass of the membrane before dust collection, mg; *q* is sampling flow rate, 30 L/min; *t* is sampling time; 1 min.

Secondly, the total dust removal efficiency can be calculated by following formula:

$$\eta =\frac{c_{1}-c_{2}}{c_{1}}\times 100$$ (13)

where *η* **is** total dust removal efficiency, %; *c*_1_ **is** total mass connection in the pre-treatment air, mg∙m^-3^; *c*_2_ **is** total mass connection in the treatment air, mg∙m^-3^.

The results for the total dust removal efficiency are listed in Table 3.

**Table 3:** Test results for the total dust removal efficiency of the simulation test unit.

	Pre-treatment Air			Treated Air			Total Dust Removal Efficiency/%
	Mass of the Membrane Before Dust Collection /mg	Mass of the Membrane After Dust Collection /mg	Total Mass Connection/mg·m^-3^	Mass of the Membrane Before Dust Collection /mg	Mass of the Membrane After Dust Collection /mg	Total Mass Connection/mg·m^-3^	
**Entry 1**	97.46	201.43	3465.67	95.76	95.82	2.00	99.94
**Entry 2**	94.22	209.37	3838.33	97.32	97.42	3.33	99.91
**Entry 3**	90.74	205.91	3839.00	93.84	93.90	2.00	99.95

These results indicate that the simulation test unit achieved total dust removal efficiency values of 99.94%, 99.91%, and 99.95% over three independent tests. The average total dust removal efficiency was therefore 99.93%.

### 4.4. Pulse cleaning system

#### 4.4.1. Pulse interval influence on dust removal effect

**Fig 14:**
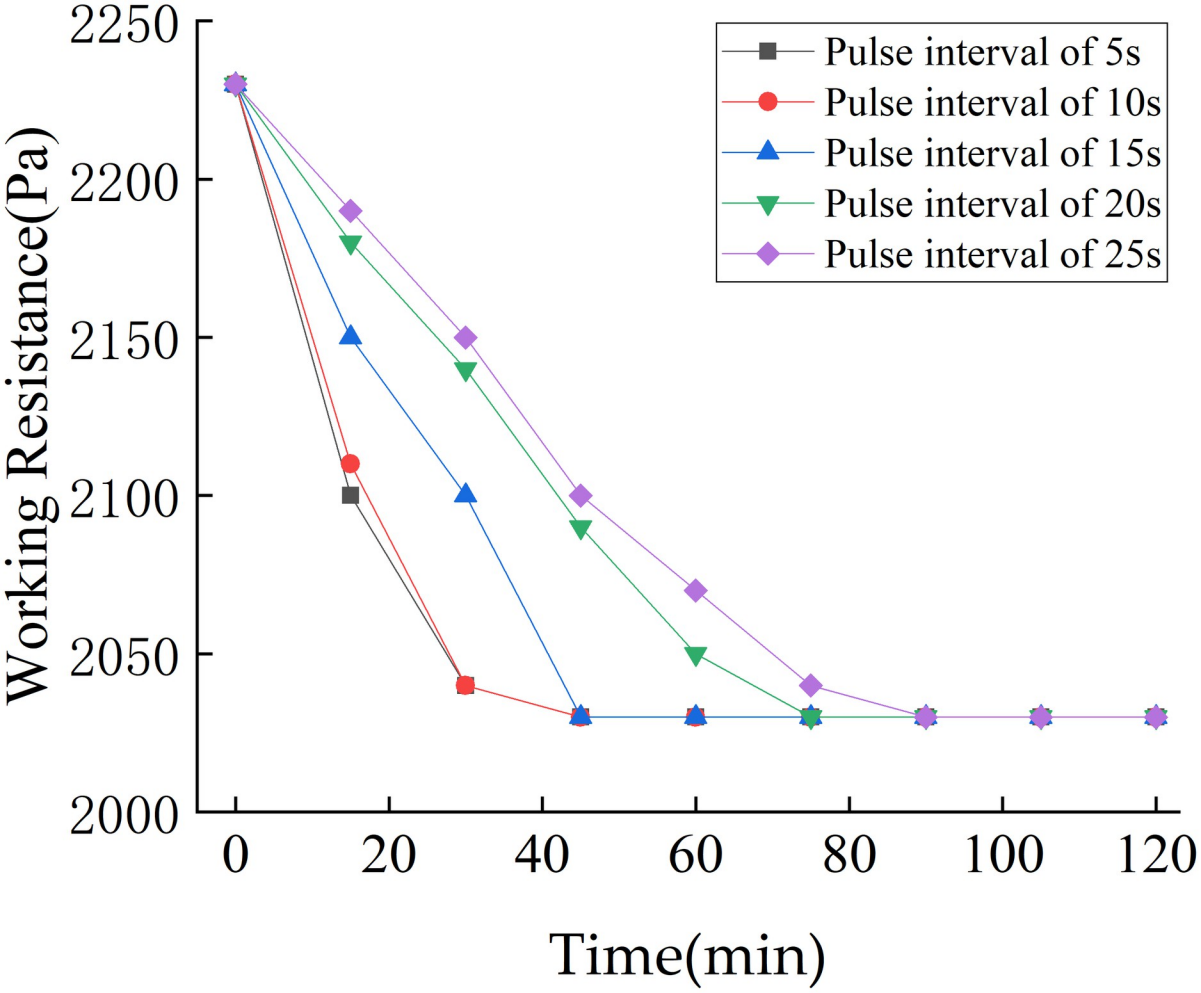
Influence of pulse interval on dust removal effect.

The compressed air pressure for the pulse cleaning system was set to 0.5 MPa and the pulse duration was set to 0.15 s. For a cycle interval of 15 s, the working resistance values at pulse intervals of 5 s, 10 s, 15 s, 20 s, and 25 s are shown in Fig 14.

As can be seen from Fig 14, a shorter pulse interval results in a shorter time required for the pulse cleaning system. When the pulse interval was 10 s, the working resistance of the simulation test unit decreased from 2230 Pa to 2030 Pa over an operational period of 45 min. The working resistance of the simulation test unit remained stable thereafter.

#### 4.4.2. Pulse duration influence on dust removal effect

**Fig 15:**
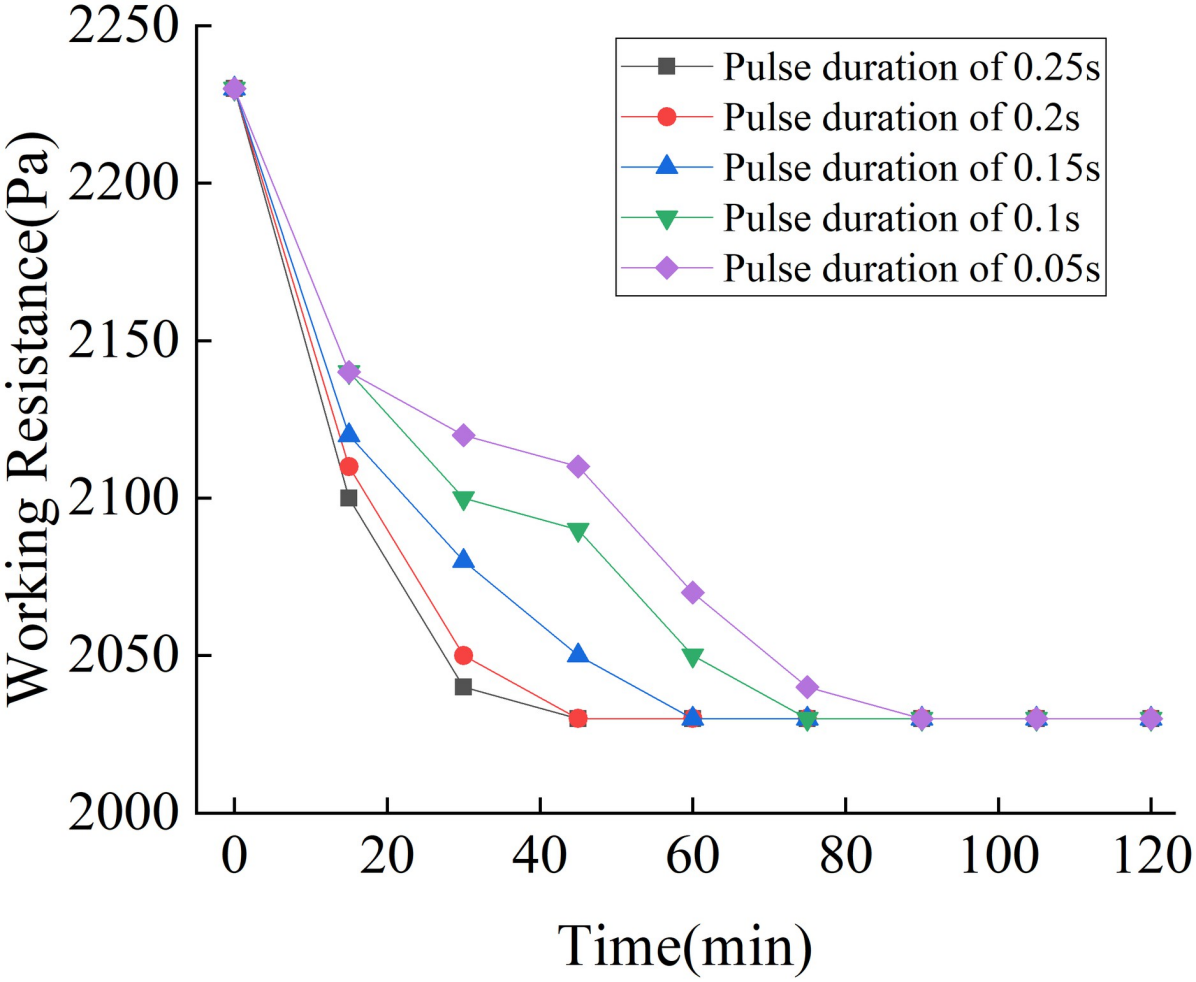
Influence of pulse duration on dust removal effect.

The compressed air pressure, pulse interval, and cycle interval of the pulse cleaning system were set to 0.5 MPa, 10 s, and 15 s, respectively, and the pulse duration was successively set to 0.05 s, 0.1 s, 0.15 s, 0.2 s, and 0.25 s. The results are shown in Fig 15.

As can be seen from Fig 15, a longer pulse duration produces a faster reduction in the working resistance of the simulation test unit. When the pulse duration was set to 0.1 s, the working resistance of the simulation test unit decreased from 2230 Pa to 2040 Pa after being operated for 45 min. The working resistance of the simulation test unit remained unchanged thereafter.

#### 4.4.3. Pulse cycle interval influence on dust removal effect

**Fig 16:**
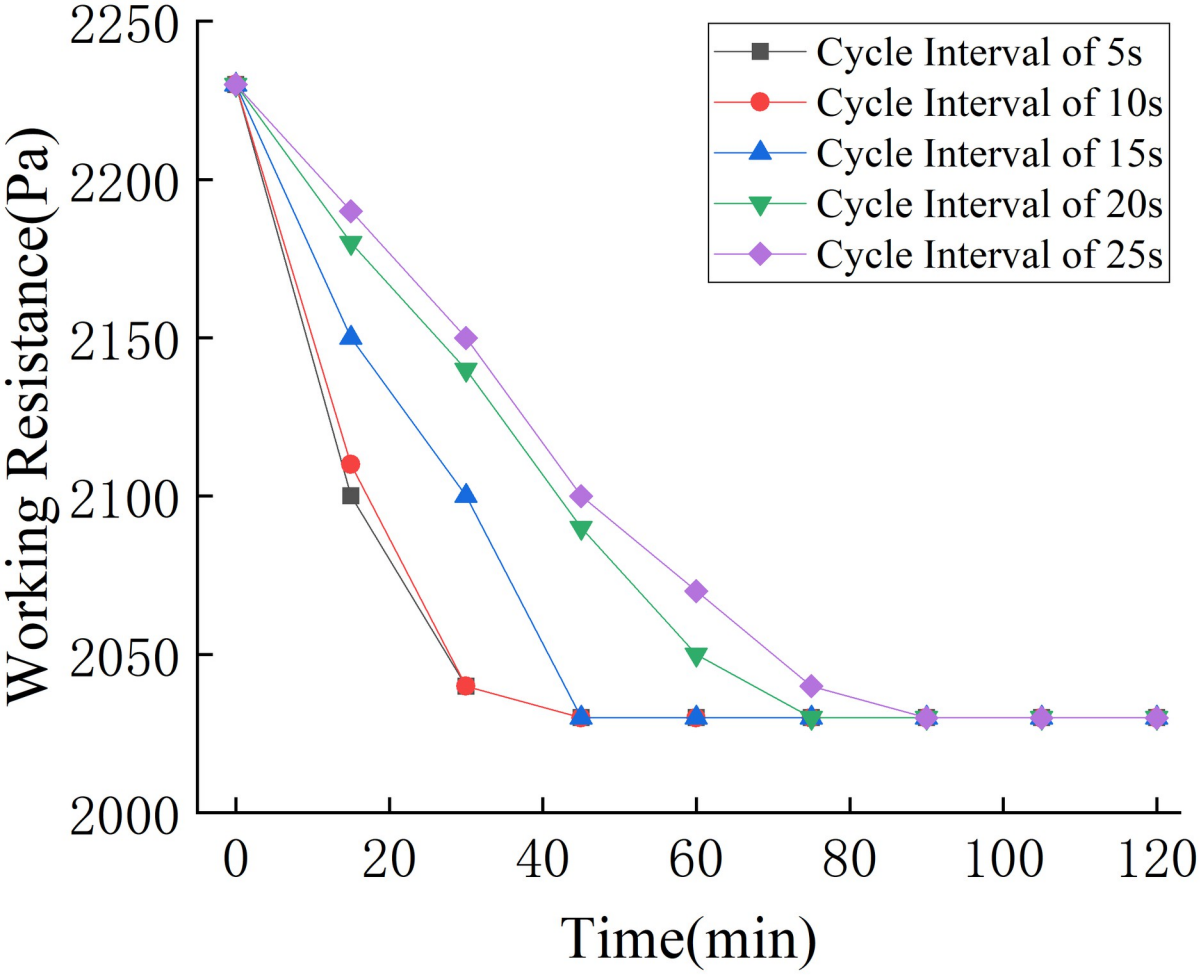
Influence of cycle interval on dust removal effect.

The compressed air pressure, pulse interval, and pulse duration for the pulse cleaning system were set to 0.5 MPa, 5 s, and 0.25 s, respectively, and the pulse cycle interval was successively set to 5 s, 10 s, 15 s, 20 s, and 25 s. The results are shown in Fig 16.

As can be seen from Fig 16, the cycle interval affects the cleaning effect. As the cycle interval increased from 5–25 s, the time taken to clear the dust in the simulation test unit increased from 45–90 min.

Considering the effect of the pulse interval, pulse duration, and cycle interval on the dust removal effect, the optimal working parameters of the pulse cleaning system were established as follows: compressed air pressure of 0.5 MPa, pulse interval of 10 s, pulse duration of 0.2 s, and cycle interval of 10 s.

## 5. Field application

### 5.1. Introduction to field applications

**Fig 17:**
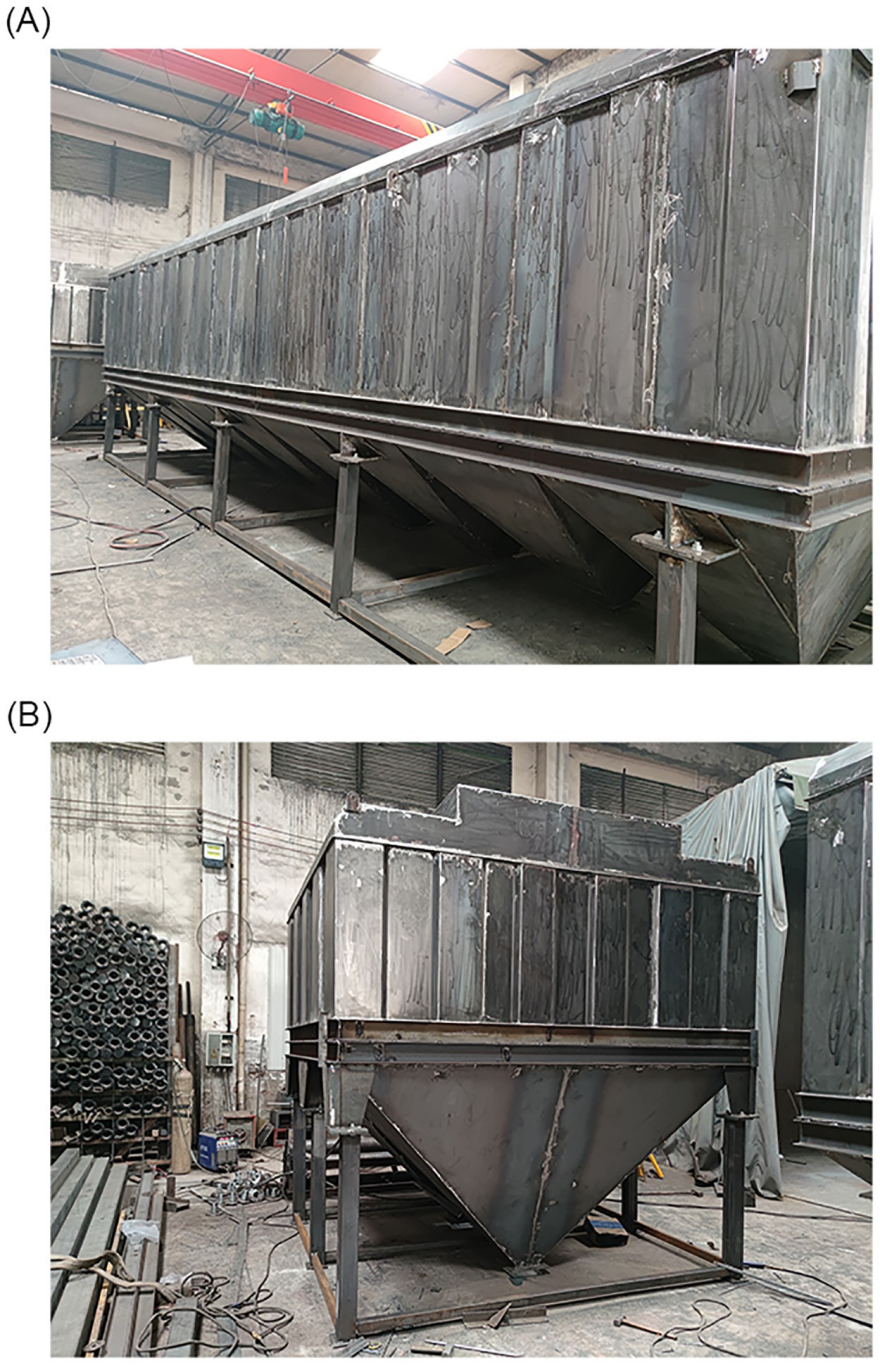
Prototype products. (A) Bag filter units of the main suction dust removal system; (B) bag filter units of the side suction dust removal system.

Bag filter units for the main and side suction dust removal systems were developed according to the numerical simulations and experimental results. The prototype products are shown in Fig 17.

**Fig 18:**
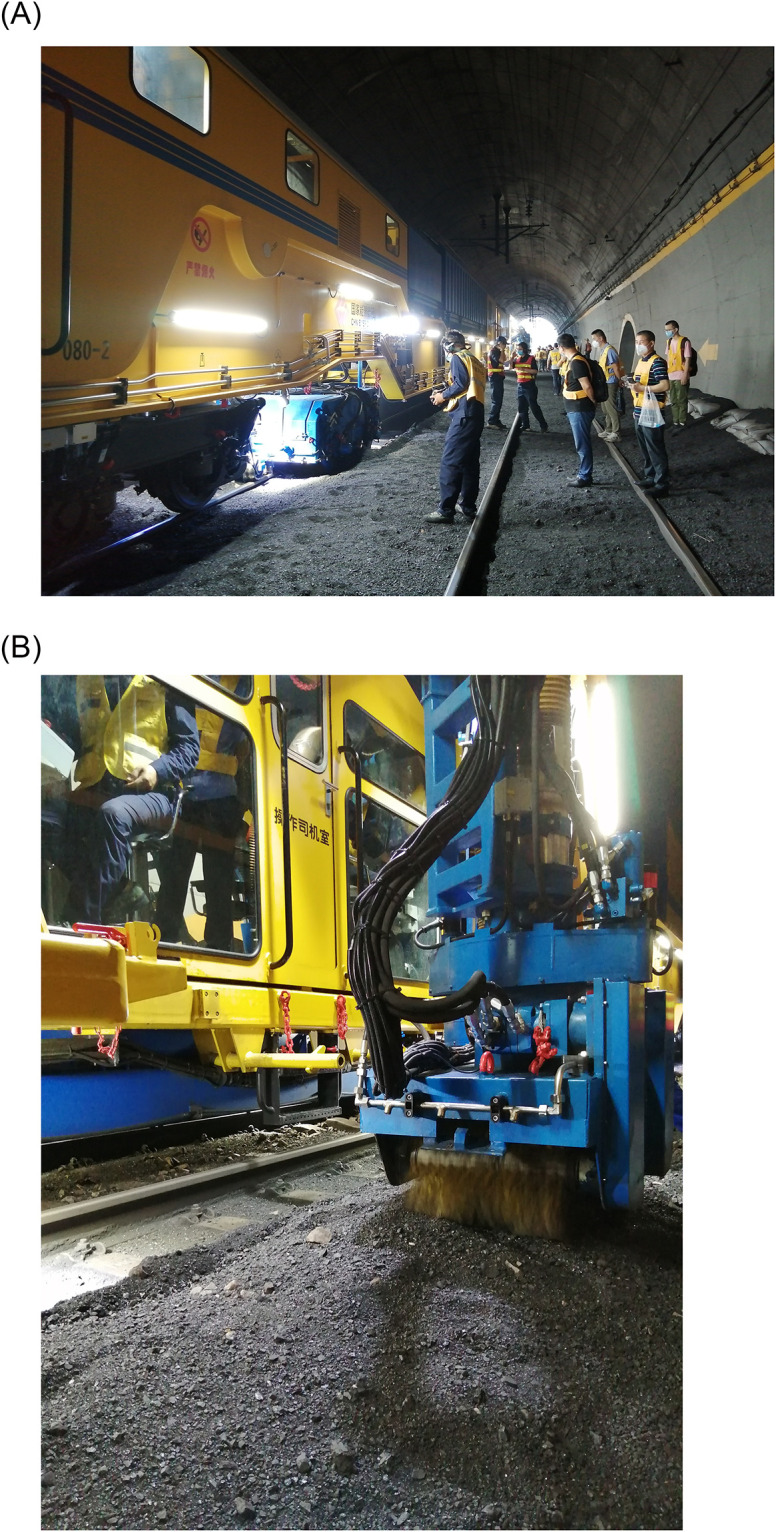
Railway ballast bed CSV in operation. (A) Main suction dust removal system in operation; (B) side suction dust removal system in operation.

A field application was carried out in Shekoumao Tunnel to investigate the application effect of the bag filter system. The railway ballast bed CSV with the updated bag filter system was operated, and the total dust mass concentration was measured at the main suction roller brush, side suction roller brush, main suction dust removal system outlet, and side suction dust removal system outlet. The total dust mass concentration was also measured at the operator position and 5 m leeward of the operator position in the tunnel. The pictures of railway ballast bed CSV in operation are shown in Fig 18.

### 5.2. Test results of application effect

The CCZ-20 respiratory dust concentration sampler was used for sampling. The sampling flow rate was set to 20 L/min and sampling was conducted for a period of 5 min. The total dust mass concentration results are presented in Table 4.

**Table 4:** Test results of total dust mass concentration.

Position	Mass of the Membrane Before Dust Collection /mg	Mass of the Membrane After Dust Collection /mg	Weight Added Value of the Membrane /mg	Total Mass Connection/mg∙m^-3^
**Main Suction Roller Brush**	68.35	68.78	0.43	4.3
**Side Suction Roller Brush**	96.84	97.11	0.27	2.7
**Main Suction Dust Removal System Outlet**	99.59	100.74	1.15	11.5
**Side Suction Dust Removal System Outlet**	73.52	74.55	1.03	10.3
**Operator Position**	75.58	76.00	0.42	4.2
**5 m Leeward of The Operator’s Position**	72.99	73.44	0.45	4.5

As can be seen from the results in Table 4, during the operation of the main and side suction dust removal systems on the railway ballast bed CSV, the total dust mass concentration did not exceed 11.5 mg∙m^−3^ at any measured position. Compared with the manual cleaning process, for which the total dust mass concentration has been measured at 335.8 mg∙m^−3^, the operating environment in the tunnel has been dramatically improved.

## 6. Conclusions

1. Numerical simulations showed that the uniformity index is 0.795 when all subchambers are open and 0.781 when subchamber 2 is closed. The uniformity index takes values of 0.812 and 0.814 under the conditions of subchamber 1 being closed and subchamber 3 being closed, respectively, indicating a slight increase in flow field uniformity. Thus, the pulse cleaning system does not significantly affect the uniformity of the flow field distribution at the bottom of the filter cartridge in the simulation test unit.
2. The experimental research showed that the original working resistance of the simulation test unit was 1990 Pa and the air volume was 30.22 m^3^·min^−1^. After the dust generator had been operating for 70 min, the working resistance of the simulation unit had increased to 2230 Pa and the air volume had been reduced to 27.42 m^3^·min^−1^. Thereafter, the working resistance and air volume remained unchanged. The simulation test unit achieved high efficiency, with an average total dust removal efficiency of 99.93%.
3. A field application showed that, during the operation of the main and suction dust removal systems on a railway ballast bed CSV, the total dust mass concentration did not exceed 11.5 mg·m^−3^ at any measured position. Compared with the manual cleaning process, which produced a total dust mass concentration of 335.8 mg·m^−3^, the operating environment in the tunnel was effectively improved.
4. The proposed system effectively improves the operational efficiency of the current bag filter system of railway ballast bed CSVs in the tunnel, effectively ensuring the operational safety of coal trains. At the same time, the proposed system significantly reduces the dust concentration in the tunnel, which is beneficial in protecting workers from dust hazards. This research provides theoretical guidance for the design of the bag filter system for railway ballast bed CSVs.

The newly developed bag filter system for railway ballast bed CSVs contributes to the efficient cleaning of coal from railway tunnels and prevents dust diffusion. The application of the bag filter system will significantly improve the working environment of cleaning personnel and protect their health. Nevertheless, the proposed bag filter system can be further optimized. First, on the premise of high dust collection efficiency, the size of the bag filter system could be further reduced. Second, the amount of compressed air used by the pulse cleaning system should be reduced as far as possible to save energy. Third, the structure of the bag filter system requires further optimization to facilitate on-site use.

## Supporting information

S1 Fig Histogram of speed values at measuring points under different conditions. (DOCX)

S2 Fig The variation of air volume and working resistance of the simulation test unit. (DOCX)

S3 Fig Test results of pulse interval influence on dust removal effect. (DOCX)

S4 Fig Test results of pulse duration influence on dust removal effect. (DOCX)

S5 Fig Test results of cycle interval influence on dust removal effect. (DOCX)

S1 Table Test results of total dust removal efficiency of the simulation test unit. (DOCX)

S2 Table Test results of total dust mass concentration. (DOCX)
