# Supplementary material for: Research and application of bag filter system for railway ballast bed coal suction vehicles: An optimization and application study
Source: PLoS One. 2024 Apr 5;19(4):e0300192. doi: 10.1371/journal.pone.0300192 (PMC10997111; doi:10.1371/journal.pone.0300192)
Supplement: S1 Fig — (DOCX) [file pone.0300192.s001.docx]

(A) (B)

(C) (D)

**S1 Fig.** **Histogram of speed values at measuring points under different conditions.** (A) All subchambers open; (B) subchamber 1 closed; (C) subchamber 2 closed; (D) subchamber 3 closed.
