# Supplementary figures and images for: Research and application of bag filter system for railway ballast bed coal suction vehicles: An optimization and application study
Source: PLoS One. 2024 Apr 5;19(4):e0300192. doi: 10.1371/journal.pone.0300192 (PMC10997111; doi:10.1371/journal.pone.0300192)

**
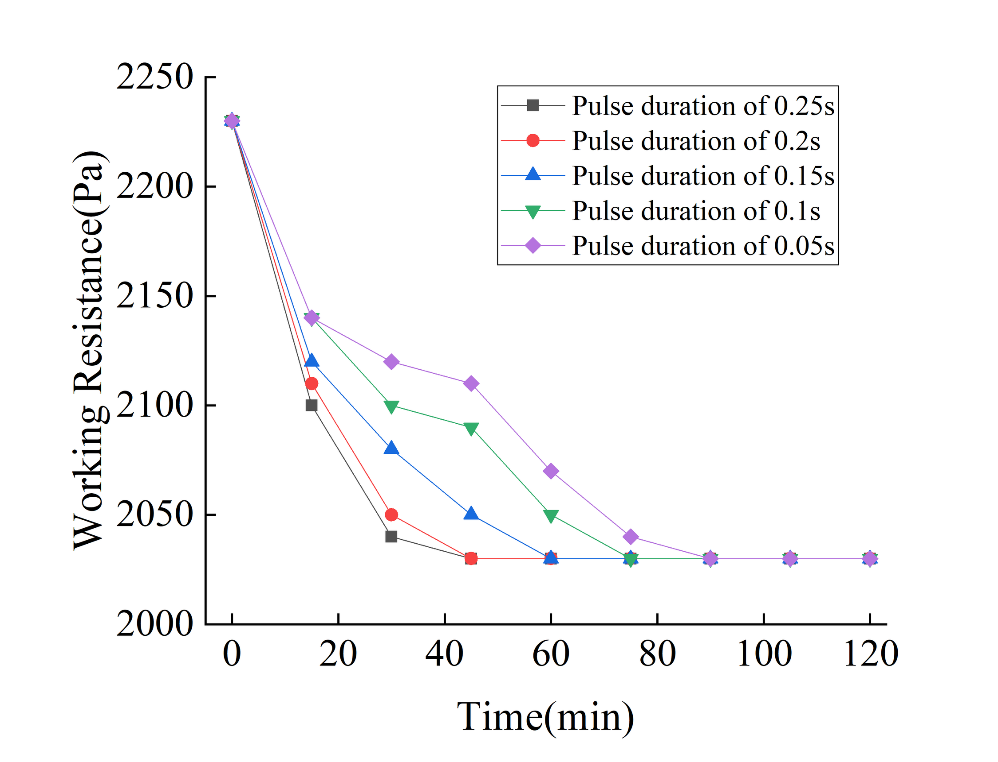
**

**S4 Fig.** **Influence of pulse duration on dust removal effect.**

Supplement: S4 Fig — (DOCX) [file pone.0300192.s004.docx]
