# Supplementary material for: Research and application of bag filter system for railway ballast bed coal suction vehicles: An optimization and application study
Source: PLoS One. 2024 Apr 5;19(4):e0300192. doi: 10.1371/journal.pone.0300192 (PMC10997111; doi:10.1371/journal.pone.0300192)
Supplement: S5 Fig — (DOCX) [file pone.0300192.s005.docx]

**S5 Fig.** **Influence of cycle interval on dust removal effect**
