# Supplementary material for: Research and application of bag filter system for railway ballast bed coal suction vehicles: An optimization and application study
Source: PLoS One. 2024 Apr 5;19(4):e0300192. doi: 10.1371/journal.pone.0300192 (PMC10997111; doi:10.1371/journal.pone.0300192)
Supplement: S1 Table — (DOCX) [file pone.0300192.s006.docx]

**S1 Table. Test results for the total dust removal efficiency of the simulation test unit.** Table showed that the simulation test unit's three total dust removal efficiency tests were 99.94%, 99.91%, and 99.95%, with an average total dust removal efficiency of 99.93%.

|  | **Pre-treatment Air** | | | **Treated Air** | | | **Total Dust Removal Efficiency/%** |
| --- | --- | --- | --- | --- | --- | --- | --- |
|  | **Mass of the Membrane Before Dust Collection /mg** | **Mass of the Membrane After Dust Collection /mg** | **Total Mass Connection/mg·m^-^³** | **Mass of the Membrane Before Dust Collection /mg** | **Mass of the Membrane After Dust Collection /mg** | **Total Mass Connection/mg·m^-^³** |  |
| **Entry 1** | 97.46 | 201.43 | 3465.67 | 95.76 | 95.82 | 2.00 | 99.94 |
| **Entry 2** | 94.22 | 209.37 | 3838.33 | 97.32 | 97.42 | 3.33 | 99.91 |
| **Entry 3** | 90.74 | 205.91 | 3839.00 | 93.84 | 93.90 | 2.00 | 99.95 |
