# Supplementary material for: Research and application of bag filter system for railway ballast bed coal suction vehicles: An optimization and application study
Source: PLoS One. 2024 Apr 5;19(4):e0300192. doi: 10.1371/journal.pone.0300192 (PMC10997111; doi:10.1371/journal.pone.0300192)
Supplement: S2 Table — (DOCX) [file pone.0300192.s007.docx]

**S2 Table. Test results of total dust mass concentration.**

| Position | Mass of the Membrane Before Dust Collection /mg | Mass of the Membrane After Dust Collection /mg | Weight Added Value of the Membrane /mg | Total Mass Connection/mg•m-³ |
| --- | --- | --- | --- | --- |
| **Main Suction Roller Brush** | 68.35 | 68.78 | 0.43 | 4.3 |
| **Side Suction Roller Brush** | 96.84 | 97.11 | 0.27 | 2.7 |
| **Main Suction Dust Removal System Outlet** | 99.59 | 100.74 | 1.15 | 11.5 |
| **Side Suction Dust Removal System Outlet** | 73.52 | 74.55 | 1.03 | 10.3 |
| **Operator Position** | 75.58 | 76.00 | 0.42 | 4.2 |
| **5 Meters Leeward of The Operator's Position** | 72.99 | 73.44 | 0.45 | 4.5 |
